# Exercise-Induced Norepinephrine Decreases Circulating Hematopoietic Stem and Progenitor Cell Colony-Forming Capacity

**DOI:** 10.1371/journal.pone.0106120

**Published:** 2014-09-02

**Authors:** Julia M. Kröpfl, Ingeborg Stelzer, Harald Mangge, Karin Pekovits, Robert Fuchs, Nathalie Allard, Lukas Schinagl, Peter Hofmann, Gottfried Dohr, Sandra Wallner-Liebmann, Wolfgang Domej, Wolfram Müller

**Affiliations:** 1 Institute of Human Movement Sciences and Sport, Exercise Physiology Lab, ETH Zurich, Zurich, Switzerland; 2 Institute of Biophysics, Medical University of Graz, Graz, Austria; 3 Clinical Institute of Medical and Chemical Laboratory Diagnostics, Research Unit on Lifestyle and Inflammation-associated Risk Biomarkers, Medical University of Graz, Graz, Austria; 4 Department of Ophthalmology, Medical University of Graz, Graz, Austria; 5 Institute for Pathophysiology and Immunology, Medical University of Graz, Graz, Austria; 6 BioTechMed-Graz, Karl-Franzens University & Technical University & Medical University of Graz, Graz, Austria; 7 Institute of Sports Science, Karl-Franzens University of Graz, Graz, Austria; 8 Institute of Cell Biology, Histology and Embryology, Medical University of Graz, Graz, Austria; 9 Department of Pulmonology, Medical University of Graz, Graz, Austria; Goethe Universität Frankfurt, Germany

## Abstract

A recent study showed that ergometry increased circulating hematopoietic stem and progenitor cell (CPC) numbers, but reduced hematopoietic colony forming capacity/functionality under normoxia and normobaric hypoxia. Herein we investigated whether an exercise-induced elevated plasma free/bound norepinephrine (NE) concentration could be responsible for directly influencing CPC functionality. Venous blood was taken from ten healthy male subjects (25.3+/−4.4 yrs) before and 4 times after ergometry under normoxia and normobaric hypoxia (F_i_O_2_<0.15). The circulating hematopoietic stem and progenitor cell numbers were correlated with free/bound NE, free/bound epinephrine (EPI), cortisol (Co) and interleukin-6 (IL-6). Additionally, the influence of exercise-induced NE and blood lactate (La) on CPC functionality was analyzed in a randomly selected group of subjects (n = 6) *in vitro* under normoxia by secondary colony-forming unit granulocyte macrophage assays. Concentrations of free NE, EPI, Co and IL-6 were significantly increased post-exercise under normoxia/hypoxia. Ergometry-induced free NE concentrations found *in vivo* showed a significant impairment of CPC functionality *in vitro* under normoxia. Thus, ergometry-induced free NE was thought to trigger CPC mobilization 10 minutes post-exercise, but as previously shown impairs CPC proliferative capacity/functionality at the same time. The obtained results suggest that an ergometry-induced free NE concentration has a direct negative effect on CPC functionality. Cortisol may further influence CPC dynamics and functionality.

## Introduction

Circulating hematopoietic stem and progenitor cells (CPCs) are rare in human peripheral blood. Nevertheless, CPC numbers can increase under special conditions such as exercise-induced physical stress [1], [2], inflammation [3] and hypoxia [4]. Exercise has a complex influence on the body, making the exact mechanisms responsible for CPC mobilization and the influence on CPC functionality/proliferative capacity difficult to identify [5]. Previous studies have associated exercise-induced oxidative stress [6], cortisol (Co) [7], interleukin 6 (IL-6), growth hormones such as vascular endothelial growth factor (VEGF) [8], and granulocyte-colony stimulating factor (G-CSF) [9] with post-exercise increased peripheral CPC numbers. A possible influence of exercise-related peripheral blood (pb) free/bound norepinephrine (NE), free/bound epinephrine (EPI) and lactate (La) remains to be elucidated. No significant correlation between pb NE and CD34+ cell number has been found after cardiopulmonary exercise in humans [10]. On the contrary, NE re-uptake inhibition has been seen to positively correlate with hematopoietic stem and progenitor cell mobilization in mice [11]. A study by Chen et al. [12] demonstrated that epinephrine combined with G-CSF could induce hematopoietic stem and progenitor cell mobilization by down-regulating CXCR4/SDF-1 (CXC-motiv-chemokinrezeptor 4/stromal cell-derived factor-1). Milovanova et al. [13] found that antioxidant (thioredoxin) mediated lactate stimulation of CD34+ stem cells related to an autocrine activation loop involving hypoxia-inducible factor 1. Thus, a hypoxic environment may mediate the effects of lactate or exercise-induced IL-6 [14] on CPCs. Lactate dehydrogenase activity may further influence absolute pb CD34+ cell counts [15].

Although previous studies have investigated the influence of physical exercise on total CPC numbers by flow cytometry [1], [2], [8] and colony-forming unit (CFU) assays [2], the proliferative capacity/functionality of CPCs in humans has only been addressed in our study [6]. The important question of which strenuous exercise-induced mechanisms affect CPC functionality, however, remains open and needs further elucidation. Circulating hematopoietic stem and progenitor cell functionality means that CPCs are able to keep their proliferative capacity at a high level. This is analyzed by a secondary colony-forming unit granulocyte monocyte (CFU-GM) assay [6], [16]–[18]. Using this functional assay, we have previously shown the higher proliferative capacity of stem and progenitor cells in the bone marrow of voluntary life-long exercising rats [16]. Further, colony formation of bone marrow-derived erythroid burst-forming units (BFU-E) and CFU-GM significantly increased in endurance exercise-trained mice vs. sedentary controls [19]. Taken together, investigations of human hematopoietic colony formation are rare [2], [6] and this is the first study that also addresses the exercise-induced impact on CPC proliferative capacity/functionality [6]. Investigations of exercise-induced NE and/or blood lactate effects on CPC functionality are also missing. Only Maestroni et al. has suggested [20] that hematopoiesis may be under an α-adrenergic control, because NE added to bone marrow cultures decreased the number of CFUs in their survey.

Schraml et al. (2009) found that treatment with α- and β-adrenergic agonists resulted in the formation of reactive oxygen species (ROS) and led to a functional decline of murine hematopoietic stem and progenitor cells [17]. In a recently published study we found that CPC counts correlate with oxidative stress levels [6]. Based on these previous findings we raised the hypothesis that exercise-induced NE concentrations may have led to a direct decline of CPC proliferative capacity/functionality. Therefore, exercise-induced NE concentrations were determined *in vivo*, and the mean free NE concentrations were used *in vitro* to elucidate a possible direct negative effect on CPC proliferative capacity/functionality.

Hence, the aim of this study was

- to investigate the influence of exercise-induced blood and plasma parameters of both free and bound NE, EPI, Co, IL-6 and La on CPC numbers,
- to determine whether exercise-induced concentrations of NE and/or La directly influence CPC functionality, and,
- to elucidate the influence of an additional hypoxic stimulus (3,500 m for 3 h) on the above-named exercise-induced plasma parameters.

## Methods

### Subjects and study design

Ten healthy, athletic, male subjects (age: mean ± SD: 25.3±4.4 yrs, BMI: 22.9±1.7 kg/m^2^) were studied. Participants were excluded from the study if they took any medications, had previous or current health problems, smoked or took dietary supplements. All participants gave their written informed consent (approved by the Medical University of Graz, Austria, ethics commission EK decision number 21–126 ex 09/10). The work load equaled a standardized cycle ergometry test protocol (3 min resting phase, 40 W starting load for 3 min increasing 20 W/min until exhaustion, followed by 40 W for 3 min and 3 min resting phase) under normoxic and normobaric hypoxic conditions (F_i_O_2_<0.15 according to an altitude of 3,500 m above sea level) in a hypoxic chamber. Air temperature and humidity were the same in both conditions and CO_2_ content was held constant by continuous absorption. All tests were performed on an electronically braked cycle ergometer (Ergoline ergometrics 800s, Ergoline, Germany) at the same time of day; blood lactate concentration (La) was determined from capillary blood samples (Biosen S_line, EKF-diagnostic, Germany) taken from the hyperemized earlobe at rest, after warm-up, after every workload step and after 3 min and 6 min of recovery. There was a break of at least 7 days between normoxic and normobaric hypoxic interventions. Blood for CPC count and plasma levels of Co, La, and IL-6 for all 10 subjects was collected at rest and at 4 time points post-exercise (10 min, 30 min, 60 min, 120 min). Plasma for NE and EPI determination was isolated at rest and at 2 time points post-exercise (0 min and 10 min). A single blood collection for *in vitro* experiments (secondary CFU-GM assays was taken from a random subgroup of 6 people on a separate day after the *in vivo* study.

Absolute CPC and blood cell counts from the study have already been published [6]. The present manuscript addresses important original (unpublished) data from the same study design and additional *in vitro* results obtained afterwards. It has to be noted that the *in vitro* assays selected to underline our hypothesis took too long to be able to publish all the results at once. For the present dataset, subjects were the same as in the *in vivo* study (n = 10) [6] and *in vitro* assays were done on a randomly selected subgroup of 6 subjects.

### Blood sample processing

Blood from the cubital vein was collected in lithium-heparinized tubes to test for levels of IL-6 as well as Co; and in ammonium-heparinized tubes for peripheral blood mononuclear cell (PMNC) isolation and stem and progenitor cell functionality tests (secondary CFU assays); ammonium-heparinized tubes, containing 1.25 mg gluthatione, were used for catecholamine detection. Blood samples were centrifuged (∼1500×g, 10 min) and plasma was immediately frozen and stored at −80°C until analysis.

### Total blood cell counts and CD34+/CD45dim flow cytometry characterization

For total blood cell counts, samples were kept at room temperature until analysis (Sysmex, BD Biosciences). CD34PE/CD45FITC (BD Biosciences) antibody staining and flow cytometry characterization of CD34+/CD45dim side scatter low cells were done using a standard protocol [21].

### *In vitro* CFU-GM assays

The influence of exercise-induced stress parameter NE and maximal blood lactate concentration (La_max_) was evaluated *in vitro* under normoxic conditions. The *in vivo* study design was modeled by incubating isolated mononuclear cells from a randomly chosen subgroup of six subjects with both norepinephrine (Sigma-Aldrich) and lactate (Sigma-Aldrich) separately and together. Stress parameter concentrations were taken from the *in vivo* study results (free NE: 5*10^−9^ mol/l baseline, 5*10^−8^ mol/l directly after the intervention; La: 0.01 mmol/l baseline, 12 mmol/l directly after the intervention) and tested in an *in vitro* assay for CPC functionality (secondary CFU-GM assay), where either NE separately or NE plus La were added once at the beginning of incubation. The medium was not changed until colonies were plucked for addressing cell colony-forming capacity/functionality.

### Peripheral blood mononuclear cell preparation for secondary colony-forming unit- granulocyte macrophage (CFU-GM) assays

Six randomly selected *in vivo* study participants [6] gave their written informed consent for another single blood withdrawal. Peripheral blood mononuclear cells (PMNC) were obtained by standard Ficoll gradient centrifugation (Histopaque; Sigma-Aldrich) according to the manufacturer's instructions using 5–8 ml of heparin-anticoagulated blood.

### Secondary colony-forming unit granulocyte macrophage (CFU-GM) assay ( =  CPC functionality/proliferative capacity)

Mononuclear cells were plated at a concentration of 2×, 3× and 4×10^5^ cells/ml in 500 µl methylcellulose culture medium (Methocult H4534, StemCell Technologies, Vancouver, Canada) in 12-well, flat-bottom, suspension culture plates (Greiner Bio One, Kremsmünster, Austria) and incubated at 37°C in a humidified atmosphere containing 5% CO_2_ for 8 days. Then, colonies consisting of more than 40 cells were individually plucked from the methylcellulose culture medium. Each single colony (90 x) was transferred to a separate well of a 48-well flat-bottom microtitre plate, dispersed in alpha medium (Gibco) supplemented with 15% FBS and thoroughly mixed with methylcellulose culture medium to get a single cell suspension [18]. After 14–16 days, each well was scored again for the presence and number of CFU-GM colonies consisting of more than 40 cells ( =  secondary CFU-GM). The ability to form secondary colonies was expressed as area under the curve (AUC) and gave information about the secondary replating capacity, which correlates with the proliferative capacity of myeloid progenitor cells and therefore represents CPC functionality [6], [18], [22].

### Evaluation of plasma norepinephrine, epinephrine, cortisol and IL-6

For NE and EPI determination, ammonium-heparin vials were used for blood sample collection, containing 1.25 mg of gluthatione per ml blood. Plasma samples were treated with ClinRepR complete kit (RECIPE, Munich, Germany), where 1.0 ml of plasma including an internal standard was transferred into the sample preparation column. In detail, catecholamines were adsorbed at aluminum oxide and isolated from the sample matrix, the plasma supernatant was removed by centrifugation. Washing steps removed further interfering co-adsorbed substances. Catecholamines were eluted from the sample preparation column and injected (40 µl) into the HPLC system. Free and bound NE and EPI concentrations were determined using an amperometrical detector (RECIPE, Munich, Germany) and specialized software (ClarityTM, DataApex, Prague, Czech Republic) [23].

Cortisol levels were evaluated by a luminescence immunoassay (Bayer, Leverkusen, Germany) [24]. Intra-assay and inter-assay variation coefficients for ELISA were below 10%.

Interleukin-6 (IL-6) was analyzed by means of an electrochemiluminescence immunoassay (Roche Diagnostics, San Francisco, CA, USA).

### Statistical Analysis

Data are reported as means +/− standard deviation (SD). Statistical analysis and plotting were done with SPSS (IBM SPSS Statistics 19) and GraphPad Prism 5. Variables were tested for normal distribution by means of the Kolmogorov-Smirnov test. Changes in blood plasma parameters and differences in CFU-GM outcomes under different NE incubations compared to a control were tested by repeated-measures ANOVA with Bonferroni posthoc comparisons, while changes in secondary colony-forming units under norepinephrine influence as well as the combined influence of NE and La compared to control groups were assessed by paired t-tests. A p-value <0.05 was considered significant.

## Results

Preliminary results described here were summarized in a doctoral thesis [5].

### Plasma parameters of cortisol and interleukin-6 and the relation to CD34+/CD45dim side scatter low cell count

**Figure 1:**
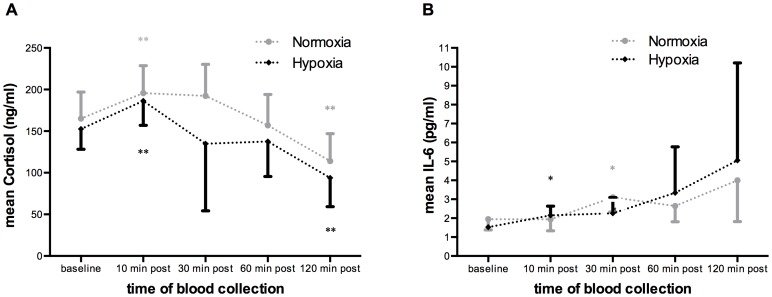
Cortisol (A) and interleukin-6 (IL-6; B) kinetics in the peripheral blood before and after an ergometry under normoxic and hypoxic conditions. Data are reported as means ± SD. Plasma cortisol significantly increased 10 min post-exercise and dropped below baseline values 120 min post-exercise under both conditions (** p<0.01, normoxia: grey, hypoxia: black). Kinetics are parallel to those of circulating hematopoietic stem and progenitor cells (CPCs) [6]. Interleukin-6 showed a time-delayed increase 30 min post-exercise under normoxia, whereas under hypoxic conditions values were already significantly elevated 10 min post-exercise. (* p<0.05, normoxia: grey, hypoxia: black).

Plasma cortisol (Co) levels showed contemporaneous kinetics as CPC counts [6], increasing significantly 10 min post-exercise and dropping significantly below baseline values 120 min post-exercise (p<0.01, Fig. 1A) under normoxic and hypoxic conditions. There was also a significant correlation between released Co levels and the difference in CPCs between time-points at normoxic conditions (r = 0.374, p<0.05). There was no significant difference between the releases of Co under normoxia vs. hypoxia throughout blood collection.

Interleukin-6 levels significantly increased above baseline time-delayed after 30 min post-exercise (p<0.05) under normoxia reaching its highest-level 120 min post-exercise (Fig. 1B). Hypoxic conditions revealed a significant increase of IL-6 levels as early as 10 min post-exercise (p<0.05), also reaching its peak-point 120 min post-exercise (Fig. 1B). Circulating hematopoietic stem and progenitor cell counts were not significantly related to IL-6 levels. There was no significant difference between the release of IL-6 levels under normoxia vs. hypoxia throughout blood collection.

### Norepinephine levels before/after exercise and the relation to CD34+/CD45dim side scatter low cell count

**Figure 2:**
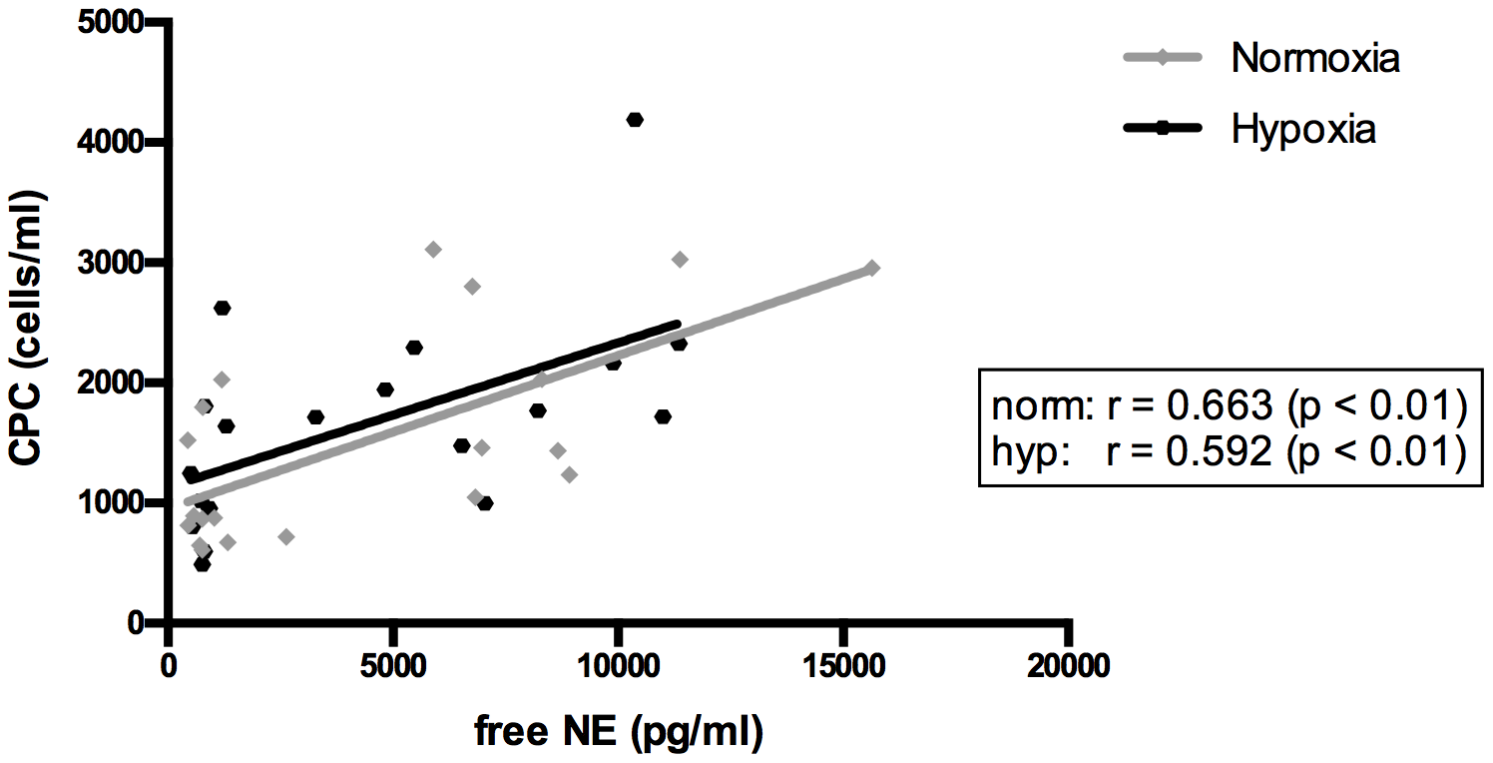
Correlation analysis of free norepinephrine (free NE) to circulating hematopoietic stem and progenitor cell count (CPC,[6]) under normoxic and hypoxic conditions (NE sampled pre, directly after; CPCs sampled pre, 10 min post; n = 20); Sampling points were the rest and peak points of the two parameters. The increase in CPCs is strongly correlated with a preceding increase in free NE.

Free norepinephrine (free NE) levels as % of total (free + bound) NE showed a significant 10-fold rise (normoxia: 32.6±12.4% to 75.1±19.5%; hypoxia: 30.8±11.1% to 77.8±15.2%, p<0.001, Table 1) immediately after the intervention under normoxia and hypoxia, whereas levels 10 min post-exercise reached a 2.5-fold increase (44.3±16.8%) under normoxic (p<0.05) and a 2.2-fold rise (49.0±15.2%) under hypoxic conditions (p<0.01). Neither were bound NE levels significantly different immediately after cessation of exercise under normoxic and hypoxic conditions (Table 1). There was no significant difference between the release of either free or bound NE levels under normoxic vs. hypoxic conditions throughout blood collection. In addition, significant positive correlations between free norepinephrine levels (pre, directly after) to CPC absolute counts (pre, 10 min post) were observed for both conditions (normoxia/hypoxia: r = 0.663/r = 0.592; p<0.01; n = 20, Fig. 2).

**Table 1:** Free/bound norepinephrine levels (pg/ml).

*Free NE*	*Baseline*	*Directly after*	*10 min post*
	*(normoxia)*	*(normoxia)*	*(normoxia)*
*Mean*	800.42	8198.71***	2006.27*
*± SD*	±299.83	±3463.23	±1130.25
	*Baseline*	*Directly after*	*10 min post*
	*(hypoxia)*	*(hypoxia)*	*(hypoxia)*
*Mean*	850.33	7794.90***	1905.70**
*± SD*	±254.67	±2805.20	±652.31

*Bound NE*	*Baseline*	*Directly after*	*10 min post*
	*(normoxia)*	*(normoxia)*	*(normoxia)*
*Mean*	2225.61	2631.93	3020.85
*± SD*	±2110.81	±2176.23	±2544.15
	*Baseline*	*Directly after*	*10 min post*
	*(hypoxia)*	*(hypoxia)*	*(hypoxia)*
*Mean*	2363.82	2323.2	2195.23
*± SD*	±1777.83	±2097.59	±1489.78

Significant differences to baseline (repeated-measures ANOVA with Bonferroni posthoc comparisons) are indicated as follows: ***p<0.001, **p<0.01, *p<0.05.

### Epinephrine levels before/after exercise and the relation to CD34+/CD45dim side scatter low cell count

Under normoxic conditions, free epinephrine levels as % of total (free + bound) EPI were significantly increased directly after cessation of exercise (40.4±25.1% to 82.7±16.0%, p<0.05) and remained different from baseline values 10 min post-exercise (44.7±24.9%, p<0.05) (Table 2). Bound epinephrine levels were significantly elevated 10 min post-exercise (p<0.05) compared to baseline, whereas under hypoxic conditions bound epinephrine levels did not reveal any significant change. Free epinephrine levels, however, also rose above baseline values immediately after cessation of exercise (46.2±19.9% to 88.5±16.4%, p<0.05) and stayed significant until 10 min post-exercise (52.0±19.6%, p<0.05) under hypoxia. There was no significant difference between the release of free/bound epinephrine levels under normoxic vs. hypoxic conditions throughout blood collection. Likewise, no significant relationship between CD34+/CD45dim cell count and free/bound epinephrine levels in the peripheral blood could be detected.

**Table 2:** Free/bound epinephrine levels (pg/ml).

*Free EPI*	*Baseline*	*Directly after*	*10 min post*
	*(normoxia)*	*(normoxia)*	*(normoxia)*
*Mean*	96.46	1219.30*	171.43*
*± SD*	±52.71	±758.72	±96.67
	*Baseline*	*Directly after*	*10 min post*
	*(hypoxia)*	*(hypoxia)*	*(hypoxia)*
*Mean*	89.48	918.22*	143.25*
*± SD*	±47.25	±542.19	±77.48

*Bound EPI*	*Baseline*	*Directly after*	*10 min post*
	*(normoxia)*	*(normoxia)*	*(normoxia)*
*Mean*	138.86	161.74	227.54*
*± SD*	±91.47	±100.48	±151.35
	*Baseline*	*Directly after*	*10 min post*
	*(hypoxia)*	*(hypoxia)*	*(hypoxia)*
*Mean*	116.10	138.70	131.36
*± SD*	±78.87	±99.73	±68.25

Significant differences to baseline (repeated-measures ANOVA with Bonferroni posthoc comparisons) are indicated as follows: *p<0.05.

### Blood lactate concentration before/after exercise and the relation to CD34+/CD45dim side scatter low cell count

Blood lactate levels were significantly increased directly after exercise cessation and during the recovery phase (3–6 min post exercise) and values significantly (normoxia: r = 0.608, p<0.01; hypoxia: r = 0.574, p<0.01) correlated with the CD34+/CD45dim cell count in the peripheral blood (La: baseline, 3–6 min post exercise, CPC: baseline, 10 min post-exercise, n = 20).

### Correlations of exercise-induced plasma parameters to blood cell counts

Under normoxic conditions, plasma cortisol (Co) showed a significant correlation with red blood cells (RBC, r = 0.474, p<0.01) as well as platelets (PLT, r = 0.344, p<0.05). Furthermore, IL-6 levels significantly correlated with platelets (r = −0.328, p<0.05) as well as neutrophils (NEU, r = 0.365, p<0.01) under normoxia. Under hypoxic conditions, cortisol levels significantly correlated with white blood cells (WBC, r = 0.312, p<0.05), red blood cells (r = 0.473, p<0.01) and lymphocytes (LYM, r = 0.420, p<0.01). Correlations for IL-6 under hypoxic conditions were significant for platelets (r = −0.345, p<0.05), neutrophils (r = 0.487, p<0.01) and lymphocytes (r = −0.301, p<0.05). A summary of reported significances can be found in Table 3.

**Table 3:** Correlations of exercise-induced plasma parameters to blood cell counts.

NORMOXIA	*Interleukin-6*	*Cortisol*
*WBC*	*n.s.*	*n.s.*
*RBC*	*n.s.*	r = 0.474**
*PLT*	r = −0.328*	r = 0.344*
*LYM*	*n.s.*	*n.s.*
*NEU*	r = 0.365**	*n.s.*

HYPOXIA	*Interleukin-6*	*Cortisol*
*WBC*	*n.s*	r = 0.312*
*RBC*	*n.s.*	r = 0.473**
*PLT*	r = −0.345*	*n.s.*
*LYM*	r = −0.301*	r = 0.420**
*NEU*	r = 0.487**	*n.s*

Pearson's correlation analysis; Pearson's r and the according p-values are reported. Abbreviations: WBC, white blood cells; RBC, red blood cells; PLT, platelets; LYM, lymphocytes; NEU, neutrophils; significances are indicated as follows: **p<0.01, *p<0.05

### Influence of exercise-induced plasma concentrations of NE and/or blood lactate on CPC functionality *in vitro*

**Figure 3:**
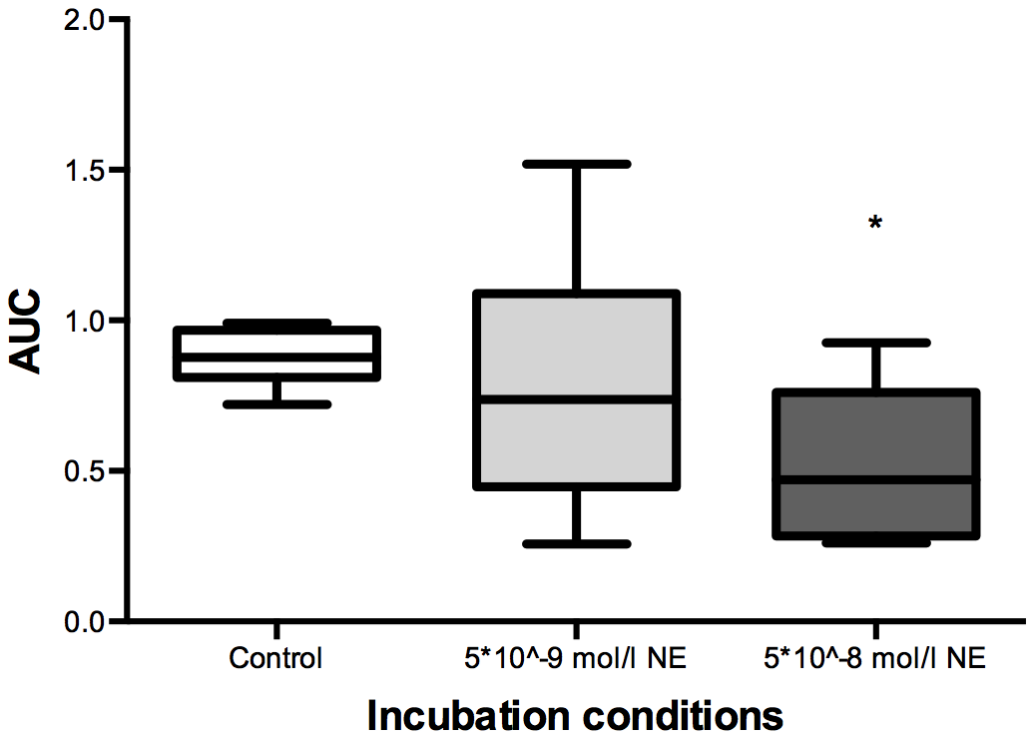
Box-plot statistics (n = 6) of the *in vitro* influence of ergometry-induced norepinephrine (NE) concentrations (5*10^−8^ mol/l) on circulating hematopoietic stem and progenitor cell (CPC) proliferative capacity/functionality compared to NE resting values (5*10^−9^ mol/l) and a control group (no NE); data are expressed as area under the curve (AUC). CPC functionality was significantly decreased under ergometry-induced NE concentrations when compared to resting values (* p<0.05). No significant change was seen comparing resting NE concentrations and a control group.

**Figure 4:**
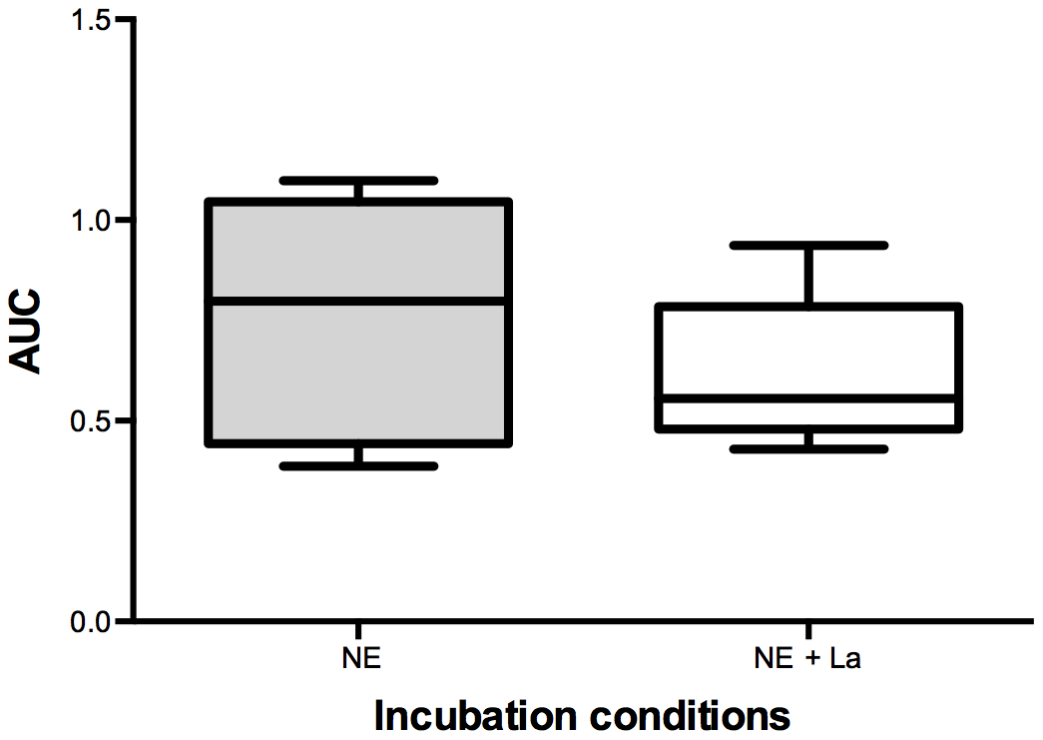
Box-plot statistics (n = 6) of the *in vitro* influence of ergometry-induced concentrations of norepinephrine (NE) and NE + lactate (NE: 5*10^−8^ mol/l; La: 12 mmol/l) on circulating hematopoietic stem and progenitor cell proliferative capacity/functionality expressed as area under the curve (AUC). Circulating hematopoietic stem and progenitor functionality was not significantly altered under the influence of both parameters, but a decreasing trend could be detected (p = 0.08).

The CPC functionality of *in vitro* secondary colony-forming units significantly declined as a result of incubation with an ergometry-induced concentration of 5*10^−8^ mol/l NE in comparison to the physiological condition of 5*10^−9^ mol/l and a control group (no NE) (repeated-measures ANOVA, with Bonferroni posthoc comparisons, p<0.05, Fig. 3). A physiological lactate concentration (values at rest) did not show any significant difference when compared to a control group (no La). When simultaneously adding both substances at an ergometry-induced concentration to the cell culture (NE: 5*10^−8^ mol/l; La: 12 mmol/l), a trend of decreasing CPC functionality was detected (p = 0.08, Fig. 4).

## Discussion

To the best of our knowledge, we are the first, i) to show, after a cycle ergometry until exhaustion under normoxic and hypoxic conditions, a significant correlation of plasma free NE (Table 1), Co (Fig. 1A), and La with CPC numbers, and ii) a direct dose-dependent impact of NE on CPC functionality *in vitro*.

In the present study, mean free NE concentrations showed an approximately 10-fold increase immediately after exercise (Table 1), which correlated positively with CPC numbers, indicating a noradrenergic effect on CPCs under normoxic and hypoxic conditions (Fig. 2). Although it is known that NE increases after exercise [25], an effect on hematopoietic progenitor cell proliferative capacity remains elusive. We are the first to analyze the biologically active form [26] of free (unconjugated) NE, which is probably more relevant for an influence on CPC functionality. Benschop et al. [27] and Dar et al. [28] showed that increased catecholamines lead to elevated leucocytes and hematopoietic stem and progenitor cells (HSPCs) in peripheral blood. Accordingly, mice with reduced NE production failed to respond to HSPC mobilization by G-CSF [29]. Further, the HSPC release from bone marrow follows a circadian secretion of NE, indicating an important influence of the sympathetic system on HSPC mobilization [30]. Besides circadian rhythm, the dose and duration of catecholamine action also affects bone marrow-derived HSPC dynamics [29], [31]. Additionally, mediated by catecholamines, other factors like G-CSF influence HSPC mobilization [12].

Free epinephrine showed a 12-fold ergometry-induced increase. In contrast to free NE, there was no significant correlation of free EPI with CPC count. These different responses might be due to the fact that NE mainly binds to α1-receptors with less effect on ß-receptors. Epinephrine, however, addresses both receptor types equally [32]. Hence, our data further confirm that catecholamines not only influence the mature hematopoietic system [33], [34] but also the immature HSPC which are the source of all blood cells [35].

Further, we show that exercise-induced free NE concentrations have a direct negative impact on CPC proliferative capacity/functionality *in vitro* (Fig. 3). In a former study [17] we found that acute adrenergic stress acting through reactive oxygen species inhibits the proliferative capacity of murine HSPCs via p38/mitogen-activated protein kinase (p38/MAPK) signaling, which is in line with the results of this study and other previously published data of our group [6].

An exhaustive cycle ergometry session as was performed in this study causes significantly increased cortisol levels 10 min after cessation of exercise followed by significantly decreased baseline values 120 min post-exercise under normoxic and hypoxic conditions (Fig. 1A). Circulating hematopoietic stem and progenitor cell counts showed the same kinetics as plasma Co levels. It should be noted in this context that Co and catecholamine kinetics have been shown to be interlinked [36]. A Co induced up-regulation of T-cell subsets by the CXCR4 chemokine receptor as shown by Dimitrov et al. [36] may also be involved in CPC up-regulation [29], [37].

The interleukin-6 kinetics of our study with peak values 120 min after exercise under normoxic and hypoxic conditions (Fig. 1B) differed from the results by Moebius-Winkler et al. [8] where IL-6 levels had already begun to decline 120 min post-exercise and returned to baseline after 24 h. These differences, seen under normoxic conditions, may be explained by a delayed increase of IL-6 in the peripheral blood [38] because in this study a constant load cycling with a duration of 4 h was applied. Interleukin-6 is also linked to endothelial progenitor cell numbers, which are involved in inflammatory processes, angiogenesis and vascular remodeling [39], [40]. Although a 1,000 m all-out rowing performance did not change IL-6 plasma levels shortly after the workout [1], half-marathon and marathon distances have been shown to significantly increase IL-6 levels [7]. Interestingly, however, the number of CD34+ cells greatly increased after the short, exhaustive exercise intervention [1], whereas neither a half marathon nor a marathon changed CD34+ cell numbers [7].

Neurotrophins were recently shown to regulate bone marrow stromal cell IL-6 expression through the MAPK pathway, which may alter hematopoiesis [41]. Thus, an indirect effect of IL-6 on HSPC mobilization may be possible. It should be noted in this context that central and peripheral catecholamines also led to elevated IL-6 [42], .

To summarize, our findings support the hypothesis that the influence of exercise on HSPC mobilization and functionality is triggered by exercise-induced catecholamines. Cortisol and IL-6 may play an additional role. Both NE and Co appear to mobilize hematopoietic stem and progenitor cells into the circulation. Cortisol levels may additionally affect the bone marrow microenvironment in terms of influence on differentiation and increased mobilization of HSPCs through Notch signaling pathways which also leads to an increased homing [44], [45]. This could also explain the simultaneously increased number and reduced functionality of circulating hematopoietic stem and progenitor cells as we found in an earlier *in vivo* study [6].

Furthermore, an additional hypoxic stimulus of 3,500 m for 3 h did not show any significant effect on plasma parameters of NE, EPI, Co, IL-6 and La.

Regarding the determination of cell functionality, a limitation of our study is that limiting dilution transplant experiments are considered to be a gold-standard of stem cell quality analysis. However, this approach was not possible for this experimental design for ethical reasons, as stated previously [6].

## Conclusions

To the best of our knowledge, this is the first study that links exercise-induced stress to the number AND proliferative capacity/functionality of circulating hematopoietic stem and progenitor cells supported by functional *in vitro* assays. Since exercise activates the sympathetic nervous system and the hypothalamic-pituitary-adrenal axis, exercise is considered to be a stress model [25]. We have clearly shown that ergometry-induced plasma levels of free NE play a crucial role for hematopoietic stem and progenitor cell mobilization and that post-exercise free NE has a direct negative effect on CPC proliferative capacity/functionality. These results underline the need for precautions in future applications of exercise and regenerative medicine. Optimized training protocols may be beneficial for tissue renewal and patient recovery [46], [47].
